# Akt1-mediated Gata3 phosphorylation controls the repression of IFNγ in memory-type Th2 cells

**DOI:** 10.1038/ncomms11289

**Published:** 2016-04-07

**Authors:** Hiroyuki Hosokawa, Tomoaki Tanaka, Yusuke Endo, Miki Kato, Kenta Shinoda, Akane Suzuki, Shinichiro Motohashi, Masaki Matsumoto, Keiichi I. Nakayama, Toshinori Nakayama

**Affiliations:** 1 Department of Immunology, Graduate School of Medicine, Chiba University, 1-8-1 Inohana, Chuo-ku, Chiba 260-8670, Japan; 2 Department of Clinical Cell Biology & Medicine, Graduate School of Medicine, Chiba University, 1-8-1 Inohana, Chuo-ku, Chiba 260-8670, Japan; 3 Division of Endocrinology and Metabolism, Graduate School of Medicine, Chiba University, 1-8-1 Inohana, Chuo-ku, Chiba 260-8670, Japan; 4 AMED-CREST, AMED, 1-8-1 Inohana, Chuo-ku, Chiba 260-8670, Japan; 5 Department of Medical Immunology, Graduate School of Medicine, Chiba University, 1-8-1 Inohana, Chuo-ku, Chiba 260-8670, Japan; 6 Department of Molecular and Cellular Biology, Medical Institute of Bioregulation, Kyushu University, 3-1-1 Maidashi, Higashi-ku, Fukuoka 812-8582, Japan

## Abstract

Th2 cells produce Th2 cytokines such as IL-4, IL-5 and IL-13, but repress Th1 cytokine IFNγ. Recent studies have revealed various distinct memory-type Th2 cell subsets, one of which produces a substantial amount of IFNγ in addition to Th2 cytokines, however it remains unclear precisely how these Th2 cells produce IFNγ. We herein show that phosphorylation of Gata3 at Ser308, Thr315 and Ser316 induces dissociation of a histone deacetylase Hdac2 from the Gata3/Chd4 repressive complex in Th2 cells. We also identify Akt1 as a Gata3-phosphorylating kinase, and the activation of Akt1 induces derepression of *Tbx21* and *Ifng* expression in Th2 cells. Moreover, T-bet-dependent IFNγ expression in IFNγ-producing memory Th2 cells appears to be controlled by the phosphorylation status of Gata3 in human and murine systems. Thus, this study highlights the molecular basis for posttranslational modifications of Gata3 that control the regulation of IFNγ expression in memory Th2 cells.

## Abstract (web summary)

Although IFNγ is a Th1 signature cytokine and is repressed in Th2 cells, it is reactivated in a subset of memory Th2 cells. Here the authors show that Hdac2 is released from Gata3 by Akt-mediated phosphorylation, leading to transcriptional derepression of Tbx21 and IFN_γ_ in these cells.

## Graphical abstract

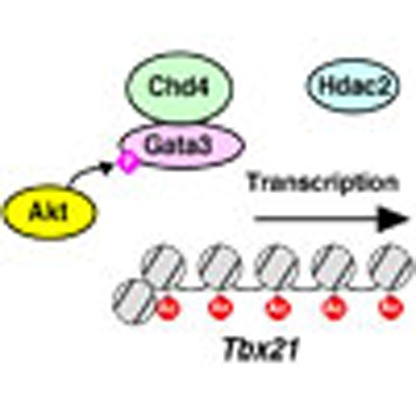

The appropriate expression of master transcription factors and effector cytokines in T helper (Th) cell subsets is essential for their immunoregulatory functions^1^,^2^. The Th2 cell differentiation programme possesses strong feed-forward mechanisms to maintain Th2 cell identity through the effector to memory phases^3^,^4^,^5^. Recent reports, however, have identified distinct memory-type Th2 cell subsets that produce a substantial amount of IL-5, IL-17 or IFNγ in addition to IL-4 and IL-13 (refs 6, 7). IFNγ production from the memory Th2 cell subset is regulated by T-bet, the master transcription factor for Th1 cell differentiation, and its expression is crucial for preventing Lymphocytic choriomeningitis virus persistence and fatal immunopathology^6^. More recently, IFNγ produced from memory T cells was shown to be essential for the mobilization and activation of innate cells and pathogen clearance^8^. However, the detailed molecular mechanisms underlying IFNγ production from Gata3-expressing memory-type Th2 cells remain unclear.

Gata3 is predominantly expressed in T lymphocytes and required for both early T-cell development in the thymus and functional differentiation of naive CD4 T cell into Th2 cells^9^,^10^,^11^. More recently, a critical role of Gata3 in group 2 innate lymphoid cell development and function was reported^12^. In peripheral CD4 T cells, IL-4-dependent activation of STAT6 induces the upregulation of Gata3 transcription^13^,^14^,^15^. In addition, the Ras-ERK MAPK cascade controls Gata3 stability through the ubiquitin/proteasome-dependent pathway^16^,^17^,^18^. A high-level expression of Gata3 is necessary and sufficient for Th2 cytokine expression in CD4 T cells. Indeed, the deletion of *Gata3* in peripheral CD4 T cells prevents their differentiation into the Th2 lineage, causing cells to differentiate towards a Th1 phenotype in the absence of polarizing cytokines^19^. Conversely, the introduction of Gata3 into developing Th1 cells switches their polarity to a Th2 phenotype^20^. Gata3 exerts at least three distinct functions by forming activating and repressive complexes: Gata3 induces differentiation of naive CD4 T cells into Th2 cells by induction of chromatin remodelling of the Th2 cytokine loci, facilitation of Th2 cell proliferation, and inhibition of Th1 cell differentiation via repression of *Tbx21*, encoding T-bet^4^,^11^,^21^,^22^. However, a molecular switch for organizing the distinct Gata3 complexes in Th2 cells has not yet been identified.

The function of transcription factors is regulated by several different posttranslational modifications, including acetylation, ubiquitination, sumoylation, methylation and phosphorylation^23^. Among them, phosphorylation has been suggested to affect strongly the function of GATA factors. Phosphorylation of Gata1 at Ser310 and Ser26 is required for erythroid differentiation and attenuation of the colony-forming activity of erythrocyte-committed progenitors, respectively^24^,^25^. Insulin-dependent phosphorylation at Ser401 of Gata2 impairs Gata2 translocation into the nucleus and its DNA-binding activity during adipocyte differentiation^26^. A recent report demonstrated the phosphorylation of Gata3 at Thr156 in thymocytes and the function of this phosphorylation in ubiquitilation and degradation of Gata3 protein^27^. However, no definitive analysis has been reported regarding phosphorylation of Gata3 and its roles in Th2 cell differentiation and function.

We herein identified that phosphorylation at Ser308, Thr315 and Ser316 in the C-finger region of Gata3 plays a critical role in the repression of T-bet-mediated IFNγ production from Th2 cells. Phosphorylation at these sites induces dissociation of Histone deacetylase 2 (Hdac2) from the Gata3/Chd4 repressive complex. We found that Akt1 is a Gata3 phosphorylating kinase for these residues, and activation of Akt1 induces derepression of *Tbx21* and *Ifng* expression in Th2 cells. In both human and murine systems, IFNγ expression in the IFNγ-producing memory-type Th2 cells appears to be regulated by the phosphorylation status of Gata3. Therefore, this study highlights the phosphorylation of Gata3 as a critical role in the repression of IFNγ production from memory-type Th2 cells through the change in the organization of the Gata3 complex.

## Results

### Phosphorylation of Gata3 induces dissociation of Hdac2

**Figure 1:**
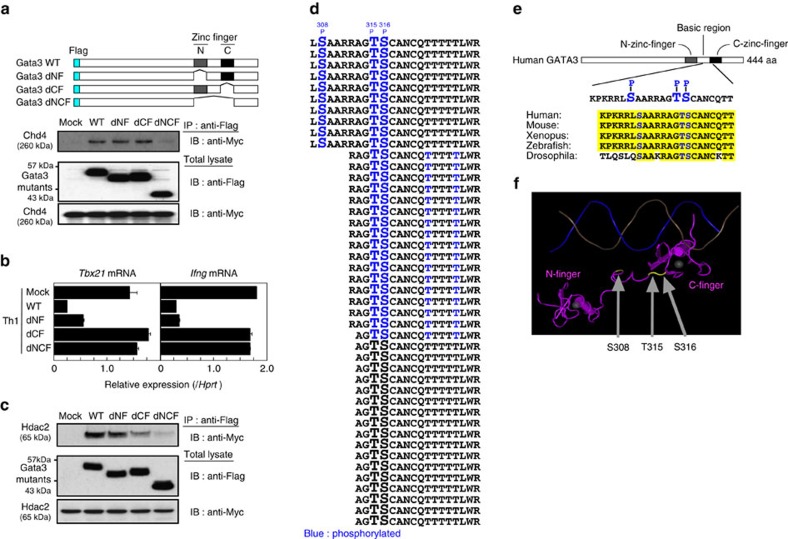
Identification of Gata3 phosphorylation in the C-terminal zinc finger. (**a**) Schematic representations of the Flag-tagged Gata3 WT or deletion mutants are shown (top panel). Flag-tagged Gata3 WT, dNF, dCF or dNCF plasmid constructs were transfected with Myc-tagged Chd4 into 293T cells. Two days later, the amount of Myc-tagged Chd4 associated with the Flag-tagged WT or mutant Gata3 was assessed by immunoprecipitation (IP) followed by immunoblotting (IB) (middle panel). Total lysates were also subjected to IB in parallel (lower panel). (**b**) Naive CD4 T cells were stimulated under Th1 conditions and then infected with a retrovirus vector carrying WT or mutant Gata3 cDNA. Three days later, the retrovirus-infected GFP-expressing cells were purified and the levels of mRNA of *Tbx21* and *Ifng* were measured by RT-qPCR. The relative expression (/*Hprt*) is shown with s.d.'s. (**c**) The amount of Myc-tagged Hdac2 associated with Flag-tagged Gata3 mutants were assessed as in Fig. 1a. (**d**) D10G4.1 cells were infected with a lentivirus encoding Flag-Gata3 and then the immunopurified Gata3 was subjected to a LC-MS/MS analysis to assess posttranslational modifications. All Gata3 peptides including Thr315 and Ser316 detected by our mass spectrometry analysis are shown. Blue characters indicate phosphorylated amino acids. (**e**) The phosphorylated residues of Gata3 in the linker region of tandem zinc fingers are highly conserved from Drosophila to human. (**f**) The 3D structure of Gata3 zinc fingers bound to DNA, including the novel phosphorylation sites (Ser308, Thr315 and Ser316) determined using the Molecular Modeling Database (MMDB ID; 105495)^30^, was drawn using the Cn3D software programme. The phosphorylated Ser/Thr residues are highlighted in yellow. Four (**b**) and three (**a**,**c**) independent experiments were performed with similar results.

We wished to identify the mechanisms by which the molecular switch for organizing activating and repressive Gata3 complexes occurs in Th2 cells. When Gata3 associates with the Chd4-NuRD repressive complex, the Gata3/Chd4-NuRD complex binds to the *Tbx21* locus and represses its expression in Th2 cells^4^. First, to determine which domains of Gata3 are important for binding to Chd4, Myc-tagged Chd4 and Flag-tagged wild type (WT) or deletion mutants of Gata3 (Fig. 1a, upper) were co-transfected into 293T cells and then pull-down assays were performed. The association with Chd4 was almost completely abrogated by the deletion of the two zinc finger domains of Gata3 (Fig. 1a), suggesting that the tandem zinc finger motifs of Gata3 are important for binding to Chd4.

Previous functional analyses showed that both the N-terminal zinc finger and C-terminal zinc finger (C-finger) motifs of Gata3, each of which is followed by a conserved basic region, are crucial for the recognition and binding to the canonical GATA consensus motif, and that the C-finger of Gata3 is required for the suppression of *Tbx21* and *Ifng* expression (Fig. 1b)^28^,^29^. In similar pull-down assays performed in Fig. 1a, we found that the C-finger of Gata3 was important for the association of Hdac2, a subunit of the NuRD complex, to the Gata3 molecule (Fig. 1c). Based on the presence of Ser/Thr clusters in the linker region as well as the basic region around the tandem zinc fingers of Gata3, we hypothesized that Gata3 phosphorylation, especially in the region necessary for the interaction between Chd4 and Hdac2, may have a role in the regulation of the bifunctional activities of the Gata3/Chd4 complex. To test this idea, we performed a liquid chromatography-tandem mass spectrometry (LC-MS/MS) analysis to identify Gata3 posttranslational modifications using immunopurified Flag-Gata3 derived from the Th2 cell clone, D10G4.1 cells. We found that Ser308, Thr315 and Ser316 in the linker region and C-finger motif of Gata3 were phosphorylated concurrently in the 10 peptides that were detected in our mass spectrometry analysis (Fig. 1d). These Gata3 residues are conserved from drosophila to human (Fig. 1e) and appear to localize to the solvent exposed region of the protein according to a crystal structure analysis of the DNA–Gata3 complex from the Molecular Modeling Database (Fig. 1f) using the Cn3D software programme^30^,^31^.

**Figure 2:**
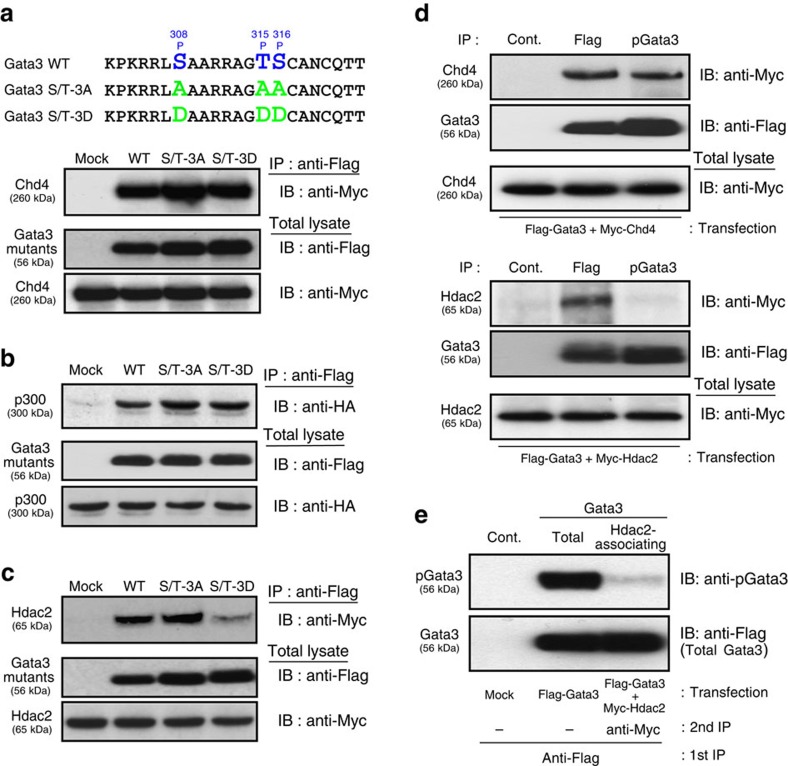
Phosphorylation of Gata3 induces dissociation of Hdac2 from the Gata3 complex. (**a**) Comparison of the amino-acid alignment of Gata3 around phosphorylated Ser/Thr residues between WT and mutants (Gata3 S/T-3A: phospho-impaired, S/T-3D: phospho-mimic) are shown (Top panel). The amounts of Myc-tagged Chd4 associated with Flag-tagged Gata3 mutants were assessed as in Fig. 1a. (**b**) The amounts of HA-tagged p300 associated with Flag-tagged Gata3 mutants were assessed. (**c**) The amounts of Myc-tagged Hdac2 associated with Flag-tagged Gata3 mutants were assessed. (**d**) Total lysates from Flag-tagged Gata3 and Myc-tagged Chd4 (upper panel) or Hdac2 (lower panel) expressing 293T cells were subjected to IP with anti-Flag mAb or anti-phospho-Gata3 Ab followed by IB with anti-Flag or anti-Myc mAb. Total lysates were also subjected to IB in parallel. (**e**) Total lysates from Flag-tagged Gata3 and Myc-tagged Hdac2-expressing 293T cells were subjected to an initial IP with anti-Flag mAb followed by a second IP with anti-Myc mAb. The immunopurified total Gata3 and Hdac2-associating Gata3 were subjected to IB with anti-phospho-Gata3 Ab or anti-Flag mAb. Four (**c**), three (**a**,**b**,**d**) and two (**e**) independent experiments were performed with similar results.

To examine the role of phosphorylation at these sites, we generated point mutants of Gata3 in which the Ser/Thr residues were substituted to an unphosphorylatable Ala and phosphate-mimic Asp (Gata3 S/T-3A and S/T-3D, respectively) (Fig. 2a, upper). 293T cells were co-transfected with Flag-tagged WT or mutants of Gata3, along with Myc-tagged Chd4, Hdac2 or HA-tagged p300, and the molecules associated with Gata3 were assessed. Interestingly, WT and mutant Gata3 proteins preserved the association with Chd4 and p300, whereas the Hdac2 association was impaired in the phospho-mimicking Gata3 S/T-3D mutant (Fig. 2a–c). Next, we used an anti-phospho-Gata3 (Ser308) antibody to address the functional role of Gata3 phosphorylation. Flag-tagged Gata3 and Myc-tagged Chd4 or Hdac2 were co-transfected into 293T cells, and then total lysates were subjected to immunoprecipitation with anti-Flag mAb or anti-phospho-Gata3 Ab. Although Chd4 association was preserved (Fig. 2d, upper), Hdac2 association was strikingly decreased in the immunoprecipitates with anti-phospho-Gata3 Ab (Fig. 2d, lower). Moreover, to assess the levels of Gata3 phosphorylation in Hdac2-associating Gata3 complexes, a two-step affinity purification using anti-Flag and anti-Myc mAbs was performed on total lysates of Flag-tagged Gata3 and Myc-tagged Hdac2 expressing 293T cells. Importantly, the Gata3 phosphorylation levels of Hdac2-associating Gata3 were substantially lower as compared with the phosphorylation levels of total Gata3 (Fig. 2e). These results indicate that phosphorylation of Gata3 at the C-finger region regulates the association of Hdac2.

### Phospho-mimic Gata3 mutant fails to repress *Ifng* expression

**Figure 3:**
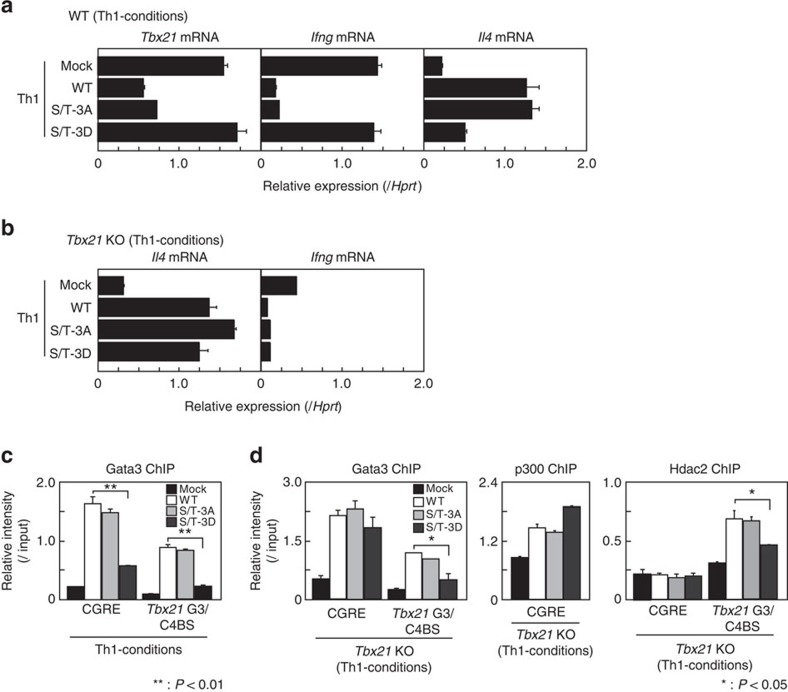
Phosphorylated Gata3 is incapable of repressing T-bet-dependent *Ifng* expression. (**a**,**b**) Naive CD4 T cells from WT (**a**) or *Tbx21*-deficient (**b**) mice were stimulated under Th1 conditions and then infected with a retrovirus vector carrying WT or mutant Gata3 (S/T-3A, S/T-3D) cDNA. Three days later, the retrovirus-infected GFP-expressing cells were purified and the levels of mRNA of *Tbx21* were measured by RT-qPCR (**a**). For the induction of cytokines, the cells were stimulated with immobilized anti-TCRβ mAb for another 4 h and then extracted RNA was subjected to RT-qPCR to assess *Il4* and *Ifng* mRNA expression (**a**,**b**). (**c**,**d**) Binding of Gata3 WT and mutants (WT, S/T-3A, S/T-3D), p300 and Hdac2 at the CGRE and the *Tbx21* G3/C4BS region was determined by ChIP assays followed by qPCR analyses in WT (**c**) or *Tbx21*-deficient (**d**) Th1 cells treated as in **a**,**b**. The mean values with s.d.'s are shown. ^**^P<0.01, *P<0.05 by Student's *t*-test. Four (**a**), three (**b**) and two (**c**,**d**) independent experiments were performed with similar results.

To assess the functional role of phosphorylation at the C-finger motif in Gata3-mediated Th2 cell differentiation, we expressed WT, S/T-3A and S/T-3D mutants in differentiating Th1 cells using a retrovirus system and examined the expression of *Tbx21, Ifng* and *Il4*. Both WT Gata3 and S/T-3A mutant Gata3 induced *Il4*, and suppressed *Tbx21* and *Ifng* expression, while the S/T-3D mutant was not able to induce *Il4* expression or repress *Tbx21* and *Ifng* expression (Fig. 3a). Given that T-bet was previously shown to interact with Gata3 directly and to repress the DNA-binding activity of Gata3 (ref. 32), we next examined the ability of Gata3 mutants to induce *Il4* expression using *Tbx21*-deficient Th1 cells to distinguish the phospho-mimic effects from the influence of T-bet-Gata3 association-dependent suppression. We found that there was no difference in the induction of *Il4* among the WT, S/T-3A and S/T-3D mutants in *Tbx21*-deficient developing Th1 cells, suggesting that the S/T-3D mutant is capable of inducing *Il4* to the same extent as WT Gata3 (Fig. 3b). In addition, the impaired repression of *Ifng* by the S/T-3D mutant in WT Th1 cells shown in Fig. 3a was not observed when *Tbx21*-deficient Th1 cells were used (Fig. 3b). These results indicate that the inability of the S/T-3D mutant to suppress IFNγ production is likely due to its inability to repress *Tbx21* expression.

We next examined the binding of Gata3 mutants to the reported Gata3-binding site (CGRE) at the Th2 cytokine loci^33^ and a Gata3/Chd4-binding site (G3/C4BS) at *Tbx21* locus^4^ by ChIP assays. The binding of the Gata3 S/T-3D mutant to the CGRE and *Tbx21* G3/C4BS regions was significantly compromised as compared with WT or S/T-3A mutant in WT differentiating Th1 cells (Fig. 3c). To avoid the direct effect of T-bet on Gata3 repression, we performed the same ChIP assays using *Tbx21*-deficent Th1 cells. The binding of the Gata3 S/T-3D mutant to the CGRE region was restored to the same level of WT or S/T-3A mutant in *Tbx21*-deficient Th1 cells, however, the binding to the *Tbx21* G3/C4BS region remained significantly decreased (Fig. 3d, left). Concomitantly, p300 recruitment to the CGRE region was unaffected in S/T-3D expressing *Tbx21*-deficient Th1 cells (Fig. 3d, middle). Furthermore, the Hdac2 association with the *Tbx21* G3/C4BS region was partially, but significantly, impaired in S/T-3D, but not in S/T-3A mutant-expressing cells (Fig. 3d, right). These results indicate that the phospho-mimic mutation of Gata3 showed decreased binding ability to the *Tbx21* locus, but had no effect on the recruitment of the Gata3/p300 transcriptional activation complex to the CGRE region. Thus, the phosphorylation status of Gata3 at the C-finger motif region appears to regulate the formation of the Gata3/Chd4-NuRD repressive complex by modulating Hdac2 association without affecting the function of the Gata3/p300 transcriptional activation complex at the CGRE region.

### Identification of Akt1 as a kinase for Gata3 phosphorylation

**Figure 4:**
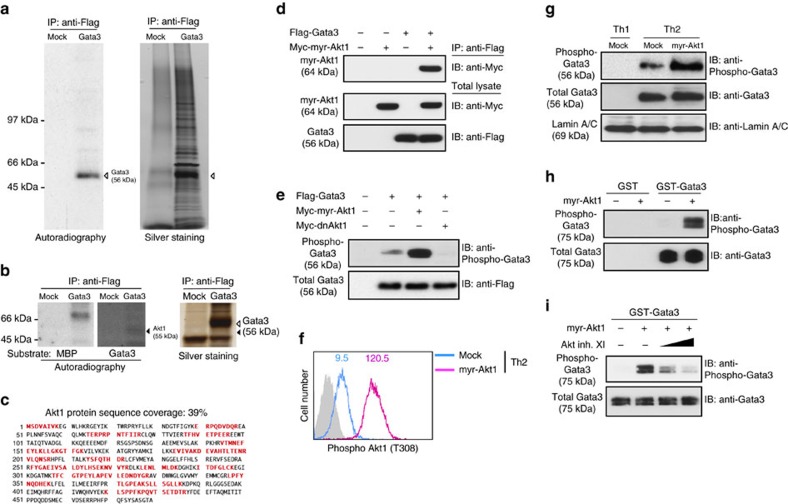
Akt1 is a kinase for Gata3 phosphorylation. (**a**) Immunopurified Gata3 complexes from 293T cells were subjected to an *in vitro* kinase assay. The white arrowheads indicate the molecular size of Gata3. (**b**) Immunopurified Gata3 complexes from 293T cells were run on a 12% SDS–PAGE gel containing recombinant Gata3 or myelin basic protein (MBP), followed by an in-gel kinase assay. Black arrowheads indicate the molecular size of Akt1. (**c**) Akt1 is detected from the band identified in Fig. 4b. Representative sequence coverage of Akt1 protein detected in a mass spectrometry analysis is shown. Red characters indicate the actual peptides detected by mass spectrometry. (**d**) The association of Myc-tagged myr-Akt1 with Flag-tagged Gata3 was assessed using 293T cells. (**e**) The amounts of phospho-Gata3 were assessed by IB with a phospho-Gata3 Ab or a Flag mAb using total lysates from Flag-tagged Gata3 and myr-Akt1 or dn Akt1-expressing 293T cells. (**f**,**g**) Naive CD4 T cells were stimulated under Th2 conditions and then infected with a retrovirus vector carrying myr-Akt1 cDNA. Four days later, intracellular staining profiles of phospho-Akt1 (Thr308) are shown. The number in the histogram represents the MFI. The grey-filled histogram shows isotype control staining (**f**). The amount of phospho-Gata3 was assessed by IB (**g**). (**h**,**i**) Recombinant Gata3 was subjected to an *in vitro* phosphorylation assay using immunopurified myr-Akt1 from 293T cells with or without Akt inhibitor XI. Five (**e**), four (**a**,**h**), three (**d**,**i**) and two (**b**,**f**,**g**) independent experiments were performed with similar results.

To identify a responsible kinase for Gata3 phosphorylation, Flag-tagged Gata3 was transfected and the Gata3 complexes were immunopurified from 293T cells and subjected to an *in vitro* kinase assay. As shown in Fig. 4a, strong ^32^P incorporation was detected at the molecular size of Gata3, indicating the existence of Gata3-phosphorylating kinases in Gata3-associated molecules. We next carried out an in-gel kinase assay to determine the molecular size of candidate kinases for Gata3 phosphorylation among Gata3-associated molecules. The Gata3-associated molecules were resolved on SDS–polyacrylamide gel electrophoresis (SDS–PAGE) copolymerized with GST-Gata3 protein as a substrate, and the in-gel kinase reaction was performed after denaturing and subsequent renaturing process of the protein-containing gel as described in the Methods section. As shown in Fig. 4b, Gata3-specific kinase activity was detected at around 55 kDa (black arrowhead), and Akt1 was identified from the 55-kDa band by a LC-MS/MS analysis (Fig. 4b,c). These results prompted us to test physical association between Gata3 and Akt1. Flag-tagged Gata3 and Myc-tagged constitutively active Akt1 (myr-Akt1) were co-transfected into 293T cells and a complex containing Gata3 and Akt1 was detected by anti-Flag immunoprecipitation and immunoblotting with anti-Myc mAb (Fig. 4d). The levels of Gata3 phosphorylation were increased in myr-Akt1-transfected 293T cells, but decreased in dominant-negative (dn) Akt1 (K179M)-transfected 293T cells (Fig. 4e). Introduction of myr-Akt1 induced the upregulation of Gata3 phosphorylation in parallel with the phosphorylation status of Akt1 (T308) in primary Th2 cells (Fig. 4f,g). To confirm that Akt1 is a Gata3-phosphorylating kinase, purified recombinant Gata3 was incubated with immunopurified myr-Akt1 in the absence or presence of Akt inhibitor XI. Gata3 phosphorylation was easily detected by immunopurified Akt1 and its phosphorylation was suppressed by the Akt inhibitor in a dose-dependent manner (Fig. 4h,i). Taken together, these results indicate that Akt1 is one of responsible kinases for Gata3 phosphorylation.

### Akt1 activation induces the derepression of IFNγ expression

**Figure 5:**
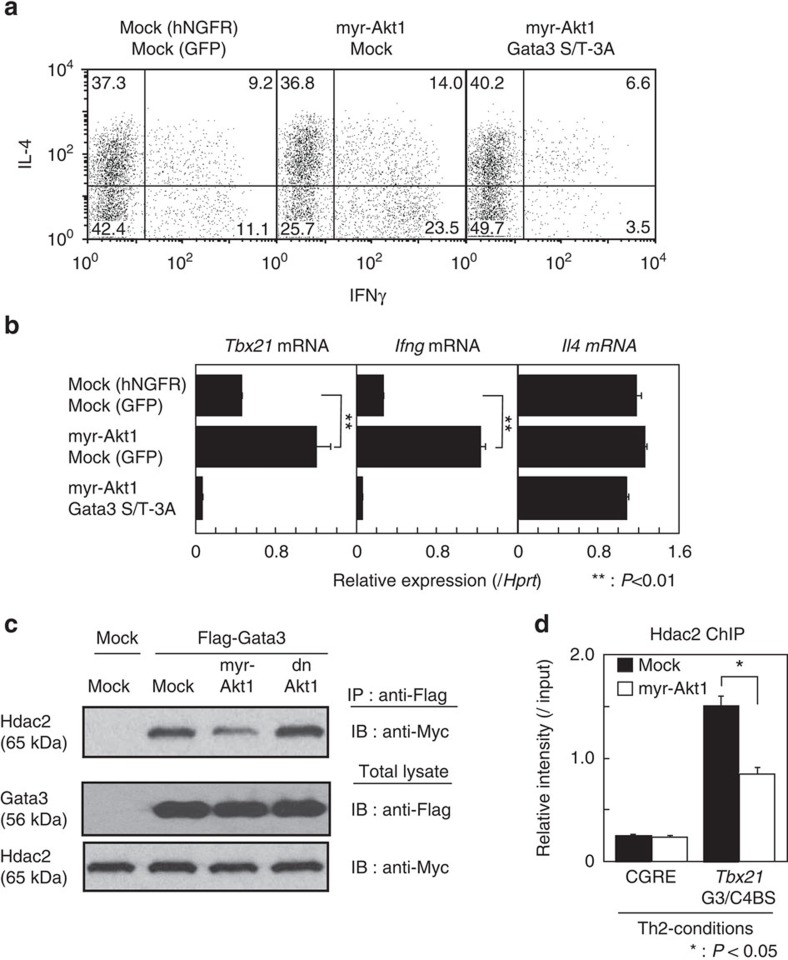
Activation of Akt1 induces the derepression of the *Tbx21* and *Ifng* expression in Th2 cells. (**a**,**b**) CD4 T cells were stimulated under Th2 conditions and then infected with retrovirus vectors carrying myr-Akt1 and Gata3 S/T-3A mutant cDNAs. Four days later, the cells were subjected to IL-4 and IFNγ staining followed by a FACS analysis. Representative IFNγ/IL-4 profiles are shown with the percentages of cells in each quadrant (**a**). The levels of mRNA of *Tbx21*, *Ifng* and *Il4* were measured by RT–qPCR (**b**). (**c**) Total lysates from Flag-tagged Gata3 and myr-Akt1 or dn Akt1-expressing 293T cells were mixed with Myc-tagged Hdac2-expressing 293T cell lysates. Then, the amounts of Myc-tagged Hdac2 associated with Flag-tagged Gata3 were assessed. (**d**) Binding of Hdac2 to the CGRE and the *Tbx21* G3/C4BS regions was determined by ChIP assays followed by qPCR analyses in myr-Akt1 introduced Th2 cells as in **a**. ^**^P<0.01, *P<0.05 by Student's *t*-test. Three (**a**,**b**) and two (**c**,**d**) independent experiments were performed with similar results.

We previously reported that inhibiting the function of the Gata3/Chd4-NuRD complex by a HDAC inhibitor, trichostatin A, induces IFNγ production from Th2 cells^4^. Thus, we sought to address whether Akt1-dependent phosphorylation of Gata3 and subsequent dissociation of Hdac2 from the Gata3 complex plays a role in the IFNγ production from Th2 cells. The active form of Akt1, myr-Akt1, was introduced into differentiating Th2 cells *in vitro*, and then the IFNγ production was assessed. The percentage of IFNγ-producing cells was increased in myr-Akt1 introduced Th2 cells (20.3% versus 37.5%), whereas no obvious effect was observed in the percentage of IL-4-producing cells (46.5% versus 50.8%) (Fig. 5a, left and middle). In addition, the introduction of the unphosphorylatable Gata3 S/T-3A mutant repressed the myr-Akt1-mediated upregulation of IFNγ production in Th2 cells (Fig. 5a, right). In accordance with the induction of IFNγ production, mRNA transcripts of *Tbx21* and *Ifng* were increased in myr-Akt1-introduced Th2 cells and the upregulation of *Tbx21* and *Ifng* mRNA mediated by myr-Akt1 was strongly repressed in Th2 cells in which the Gata3 S/T-3A mutant was introduced (Fig. 5b). Flag-tagged Gata3 and myr-Akt1 or dominant-negative Akt1 (dn Akt1) were co-transfected into 293T cells and the ability of Gata3 to associate with Myc-tagged Hdac2 was assessed by immunoprecipitation experiments. The levels of Hdac2 association with Gata3 were decreased in myr-Akt1-transfected cells, but not in dn Akt1-transfected cells (Fig. 5c). Moreover, the binding of Hdac2 to the *Tbx21* G3/C4BS region was decreased in myr-Akt1-introduced Th2 cells (Fig. 5d). These results indicate that the activation of Akt1 regulates the dissociation of Hdac2 from the Gata3 complex and the derepression of *Tbx21* and *Ifng* expression in Th2 cells.

### T-bet-dependent IFNγ production from memory Th2 cells

**Figure 6:**
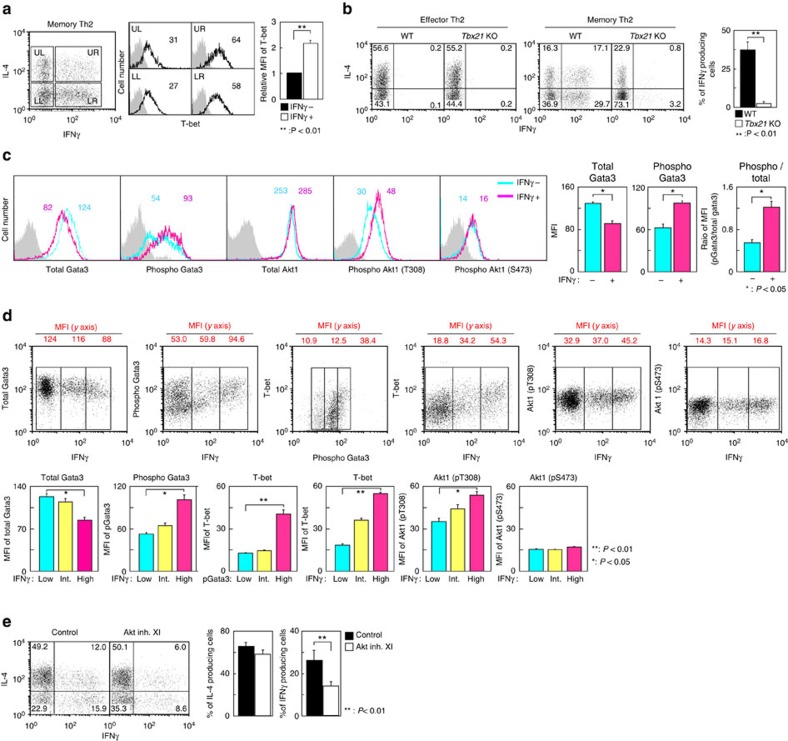
Increased phosphorylation status of Gata3 and Akt1 in IFNγ-producing memory Th2 cells. (**a**) Memory Th2 cells were stimulated with PMA plus ionomycin for 6 h. The cells were divided into four distinct subpopulations according to their expression of IL-4 and IFNγ (left). Intracellular staining profiles of T-bet in these four subpopulations are shown (middle). The number in the histogram represents the MFI. The grey-filled histogram shows isotype control staining. A summary of the MFI of T-bet in IFNγ-producing and -nonproducing cells is presented (right) (*n*=5). (**b**) Intracellular staining profiles of IFNγ and IL-4 in effector and memory Th2 cells from WT or *Tbx21*-deficient mice are shown with the percentages of cells in each area (left). A summary of the percentage of IFNγ-producing memory Th2 cells is presented (right) (*n*=3). (**c**) Intracellular staining profiles of Gata3, phospho-Gata3, Akt1 and phospho-Akt1 in IFNγ-producing and -nonproducing memory Th2 cells are shown (left). A summary of the MFI value of Gata3 and phospho-Gata3 and the ratio of the MFI (phospho-Gata3/total Gata3) in IFNγ-producing and IFNγ-nonproducing memory Th2 cells is presented (right) (*n*=5). (**d**) Intracellular staining profiles of IFNγ, Gata3, phospho-Gata3, T-bet and phospo-Akt1 are shown. The number represents the MFI of the *y* axis in each area (top). A summary of the MFI in each area (IFNγ: Low, Int, High) is presented (bottom) (*n*=5). (**e**) Memory Th2 cells were cultured in IL-7-containing medium with or without Akt inhibitor XI (10 μM) for 2 days. Intracellular staining profiles of IFNγ and IL-4 are shown (left). A summary of the percentage of IL-4- and IFNγ-producing memory Th2 cells is presented (right) (*n*=4). ^**^P<0.01, *P<0.05 by Student's *t*-test. Five (**a**), four (**e**) and three (**b**,**c**,**d**) independent experiments were performed with similar results.

Recently, distinct memory-type Th2 cell subsets have been identified in several groups, which produce a substantial amount of IL-5, IL-17 or IFNγ in addition to IL-4 and IL-13 (ref. 7). IFNγ expression in Th2 cells is reported to be dependent on T-bet and plays an important role in the protective immunity against viral infections^6^. We examined whether Gata3 phosphorylation-induced derepression of *Tbx21* expression is involved in the IFNγ production from these memory-type Th2 cells. Antigen-specific memory Th2 cells can be efficiently generated *in vivo* by adoptive transfer of effector Th2 cells^34^. To determine the expression levels of T-bet in IFNγ-producing memory Th2 cells, the cells were divided into four distinct subpopulations according to their expression of IL-4 and IFNγ. Consistent with previous report^35^, we found that IFNγ-producing memory Th2 cells express higher levels of T-bet protein as compared with non-IFNγ-producing memory Th2 cells (Fig. 6a). To examine whether IFNγ production from memory Th2 cells is T-bet-dependent, memory Th2 cells from *Tbx21*-deficient cells were generated. While there was no difference in effector Th2 cell differentiation between WT and *Tbx21*-deficient cells, IFNγ production from *Tbx21*-deficient memory Th2 cells was strongly inhibited (Fig. 6b).

### Gata3 is phosphorylated in IFNγ-producing memory Th2 cells

We next analysed the status of Gata3 phosphorylation in IFNγ-producing memory Th2 cells. Although the level of total Gata3 protein was slightly lower, the level of Gata3 phosphorylation, particularly the ratio of phosphorylated Gata3 to total Gata3 protein levels, was higher in IFNγ-producing cells compared with non-producing cells (Fig. 6c). The phosphorylation status of Akt1 at Thr308 in IFNγ-producing memory Th2 cells was higher than in non-producing cells, whereas there was only a marginal increase in the levels of total Akt1 and phosphorylated Akt1 at S473 (Fig. 6c). We also found that the phosphorylation levels of Gata3 were higher in IFNγ-producing memory Th2 cells according to the mean fluorescent intensity (MFI) value (53.0 versus 94.6) (Fig. 6d). Moreover, memory Th2 cells with higher Gata3 phosphorylation showed higher levels of T-bet expression. A similar positive correlation was detected in IFNγ-production and T-bet expression, in addition to Akt1 phosphorylation (pT308) (Fig. 6d). A summary of the MFI values with statistics was also evaluated (Fig. 6d, lower). Next, to address the role of Akt1 activation in IFNγ production from memory Th2 cells, we examined the effect of an Akt inhibitor on IL-4 and IFNγ production in memory Th2 cells. The production of IFNγ, but not IL-4, from memory Th2 cells was modestly but significantly decreased by Akt inhibitor treatment, suggesting that the production of IFNγ is regulated by Akt in memory Th2 cells (Fig. 6e). Taken together, these results indicate that Akt1 is one of the responsible kinases for the regulation of IFNγ production, potentially through Gata3 phosphorylation and subsequent derepression of the *Tbx21* expression in memory Th2 cells.

### GATA3 phosphorylation in human memory Th2 cells

**Figure 7:**
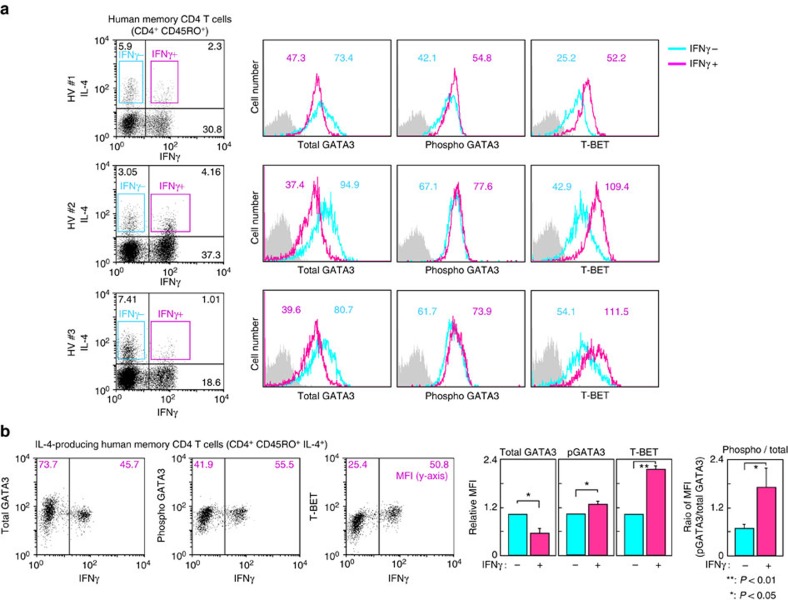
GATA3 phosphorylation in human IL-4 and IFNγ-double-producing memory-type CD4 T cells. (**a**) Human CD45RO^+^ memory CD4 T cells from peripheral blood were purified and stimulated *in vitro* for 4 h. Intracellular staining profiles of GATA3, phosopho-GATA3 and T-BET in IL-4^+^/IFNγ^−^ (IFNγ^−^) or IL-4^+^/IFNγ^+^ (IFNγ^+^) memory CD4 T cells are shown. Representative IFNγ/IL-4 profiles are shown with percentages of cells in each quadrant (left). The number in the histogram represents the MFI. The grey-filled histogram shows isotype control staining. Experiments using three healthy donor volunteers (HV#1–3) are shown. (**b**) Human CD45RO^+^ memory CD4 T cells were stimulated and the intracellular staining profiles of IFNγ, GATA3, phospho-GATA3 and T-BET are shown with the MFI of the *y* axis in each area (left). A summary of the MFI of GATA3, phospho-GATA3 and T-BET and the ratio of the MFI (phospho-GATA3/total GATA3) in IFNγ-producing and IFNγ-nonproducing memory Th2 cells is presented (right) (*n*=3). ^**^P<0.01, *P<0.05 by Student's *t*-test. Experiments using three healthy donor volunteers were performed with similar results.

Finally, we analysed memory-type Th2 cells from human peripheral blood. CD4^+^CD45RO^+^ memory CD4 T cells from healthy volunteers were isolated and stimulated *in vitro* with phorbol 12-myristate 13-acetate (PMA) and ionomycin for 4 h. In the human memory-type CD4 T cells, IL-4 single producers and IL-4/IFNγ double-producers were detected (Fig. 7a, left). Similar to murine memory Th2 cells (Fig. 6), the expression levels of total GATA3 protein were lower and IL-4/IFNγ double-producers showed higher phospho-GATA3 and T-BET protein expression as compared with IL-4 single producers (Fig. 7a). Moreover, human IL-4-producing memory-type CD4 T cells with higher levels of IFNγ expression showed higher levels of GATA3 phosphorylation and T-BET expression (Fig. 7b, left). The ratio of the MFI of phosphorylated GATA3 to the total GATA3 level was significantly higher in IFNγ-producing human memory Th2 cells as compared with IFNγ-non-producing cells (Fig. 7b, right). These results are consistent with the notion that GATA3 phosphorylation-induced derepression of T-BET expression may occur in human memory-type CD4 T cells and control the production of IFNγ.

## Discussion

Gata3 organizes functionally distinct complexes with defined functions in differentiating Th2 cells and regulates the expression of a various genes in a positive and negative fashion to establish Th2 cell identity^4^,^11^,^21^,^22^. In the current study, we wished to identify the mechanisms by which the molecular switch for organizing activating and repressive Gata3 complexes occurs in Th2 cells and found that a posttranslational modification of Gata3, phosphorylation of Gata3 mediated by Akt1, plays an important role in the organization of repressive Gata3 complexes and IFNγ expression in memory Th2 cells. Some Gata3 molecules are phosphorylated in Th2 cells and phosphorylation mimic Gata3 has a selective defect in the inhibition of IFNγ production. Phosphorylation of Gata3 at Ser308, Thr315 and Ser316 resulted in the dissociation of Hdac2 from the Gata3 complex, followed by derepression of *Tbx21* and *Ifng* expression (Figs 1, 2, 3). Akt1 was identified as a Gata3 kinase (Figs 4 and 5), and the levels of Akt1 phosphorylation and subsequent Gata3 phosphorylation were significantly upregulated in IFNγ-producing memory Th2 cells (Figs 6 and 7). This study highlights a functional link between the posttranslational modification of Gata3 and its strong feed-forward function as a master regulator of Th2 cell differentiation and function.

An array of posttranslational modifications may orchestrate the activity of a transcription factor, including subcellular localization, protein stability, sequence-specific DNA binding, transcriptional activity and protein–protein interactions^23^. Regarding the master transcription factors for Th cell subset differentiation, some pioneer studies have reported several interesting examples; Itk-mediated tyrosine phosphorylation of T-bet controls its interaction with Gata3 (ref. 32), ubiquitination of Foxp3 induced by Stub1 regulates the stability of Foxp3 protein^36^, cyclin-dependent kinase 2 (CDK2)-mediated phosphorylation of Gata3 at Thr156 regulates Fbw7-dependent Gata3 ubiquitylation and degradation in the thymus^27^, and methylation of Gata3 at Arg261 regulates the transcriptional activation of the *Il5* gene in Th2 cells^22^. We herein demonstrate that phosphorylation of Gata3 at Ser308, Thr315 and Ser316 controls the repressive function of Gata3 on IFNγ expression via the dissociation of Hdac2 from the Gata3/Chd4 repressive complex. Similar examples have been reported in p53-MDM2 systems^37^,^38^. Phosphorylation of p53 at Ser15 and Ser37 induces a significant change in p53 conformation and resulted in the impaired ability of MDM2 to inhibit p53-dependent transactivation^38^.

Akt1 phosphorylates several transcription factors, including Foxo1, CREB, Gata1 and Gata2, and regulates their functions by various distinct mechanisms^26^,^39^,^40^,^41^. Akt1-mediated phosphorylation of Foxo1 at Thr24 and Ser253 induces nuclear export and transcriptional inactivation of Foxo1 (ref. 39). CREB is phosphorylated by Akt1 at Ser133, and the phosphorylation of CREB induced targeted gene expression by prompting the recruitment of co-activator CBP^41^. Phosphorylation of Gata1 at Ser310 is induced by Akt1 and is required for the transcriptional activity of Gata1 in erythroid cells^40^. Akt1-dependent phosphorylation at Ser401 of Gata2 controls its subcellular localization in adipocytes^26^. Thus, Akt1 phosphorylates several transcription factors and positively and negatively regulates their functions. We herein demonstrate another example of negative regulation by Akt1-mediated phosphorylation: Akt1 phosphorylates Gata3 and induces the dissociation of Hdac2 from the Gata3 complex, resulting in a failure in the repression of IFNγ expression in Th2 cells.

Th2 cells are thought to be an intrinsically stable lineage that possesses a programme to produce IL-4, IL-5 and IL-13, and the machinery to inhibit the expression of IFNγ (ref. 42). However, after viral infection, previously committed Th2 cells stably co-expressing Gata3 and T-bet show the features of both Th1 and Th2 cells^6^. In addition, several investigators have reported IFNγ-producing Th2 cells under various *in vivo* and *in vitro* conditions^43^,^44^. As shown in Figs 6 and 7, memory-type Th2 cells that express IFNγ can be easily detected in both murine and human systems. Thus, the current findings of Akt1-mediated phosphorylation of Gata3, the organization of repressive Gata3 complexes and the repression of IFNγ expression may operate under physiological conditions. Memory-type Th2 cells that express IFNγ may play an important role in the pathogenesis of a mixed-type inflammation with infiltration of eosinophils and neutrophils^45^,^46^.

We found that the levels of Akt1 phosphorylation at Thr308 were substantially higher in IFNγ-producing memory Th2 cells, while there was only a marginal increase in the total Akt1 levels in IFNγ-producing cells as compared with non-producing cells (Fig. 6c). These results may indicate that the activation of Akt1 is more important than the expression of Akt1 itself in the regulation of IFNγ production in memory Th2 cells. The upstream signals that control Akt1 activity appear to be important in the selective activation of Akt1 in IFNγ-producing memory Th2 cells. We also found that the IFNγ production from memory Th2 cells was significantly inhibited by Akt inhibitor treatment (Fig. 6e). While we have not determined whether Akt1 is necessary for the generation of IFNγ-producing Th2 cells, this result may indicate that the Akt activity plays a pivotal role in the maintenance of IFNγ-producing ability in memory Th2 cells. Thus, further study is required to clarify which signals (via TCR, co-receptors or cytokine receptors) confer the selective activation of Akt1 in IFNγ-producing Th2 cells and how IFNγ-producing Th2 cells are generated *in vivo*. Further detailed analyses of the posttranslational modifications of Gata3 and identification of responsible enzymes may lead to the discovery of novel therapeutic targets for Th2-mediated, IFNγ-involved chronic inflammatory disorders, including atopic dermatitis or chronic airway inflammatory disorders.

HDR syndrome (hypoparathyroidism, deafness, renal disease) is an autosomal dominant disease characterized by the triad hypoparathyroidism, hearing loss and renal dysplasia^24^. In addition to T lymphocytes, Gata3 expression is observed in the developing parathyroid glands, inner ear and kidneys in humans and mice^47^. Loss-of-function mutations of GATA3 have been reported to be associated with HDR syndrome^24^. Interestingly, an in-frame deletion of amino acids 315–318 in GATA3 was found in patients with familial HDR syndrome. This deletion involves Cys317, which binds to zinc ions to stabilize the zinc finger structure. Since phosphorylation of GATA3 at Thr315 and Ser316 modulates the function of GATA3, it is of particular interest to study the pathophysiological roles of GATA3 phosphorylation in HDR syndrome.

In summary, Akt1-mediated phosphorylation of Gata3 plays an important role in the organization of repressive Gata3 complexes and IFNγ repression in Th2 cells. The findings presented herein highlight a pivotal role of posttranslational modification of Gata3 in the organization of Gata3 complexes and its strong feed-forward function as a master regulator of Th2 cell differentiation and function.

## Methods

### Mice

All of experiments were performed using 6–8-week-old female mice. C57BL/6, BALB/c and BALB/c *nu/nu* mice were purchased from CLEA Co. (Tokyo, Japan). OVA-specific TCR-αβ (DO11.10) transgenic (Tg) mice (BALB/c background) were provided by Dr D. Loh (Washington University School of Medicine, St Louis). *Tbx21*-deficient mice (DO11.10 Tg, BALB/c background) were kindly provided by Dr Laurie Glimcher (Cornell University). All mice were maintained under specific pathogen-free conditions. Animal care was conducted in accordance with the guidelines of Chiba University.

### Generation of Th1 and Th2 cells

CD4 T cells with a naive phenotype (CD44^low^) were purified using a FACSAria machine (Becton Dickinson, San Jose, CA) yielding a purity of >98% and stimulated with 3 μg ml^−1^ of immobilized anti-TCRβ mAb plus 1 μg ml^−1^ anti-CD28 mAb under Th1 or Th2 conditions *in vitro*. The cells were then transferred to new dishes and cultured for another 3–4 days in the presence of only the cytokines present in the initial culture. Th1 conditions were as follows: 25 U ml^−1^ of IL-2, 10 U ml^−1^ of IL-12 and an anti-IL-4 mAb. Th2 conditions were as follows: 25 U ml^−1^ IL-2, 10 U ml^−1^ IL-4, and anti-IL12 and anti-IFNγ mAbs.

### Generation of memory Th2 cells

Naive CD4 T cells from DO11.10 Tg mice were cultured with 300 nM OVA peptide (Loh15, OVA 323-339) and Thy1.2-depleted BALB/c irradiated spleen cells for 6 days under Th2 conditions. Effector Th2 cells (3 × 10^7^) were then transferred intravenously to syngeneic BALB/c *nu/nu* mice. Five weeks after cell transfer, CD4^+^ and KJ1^+^ cells in the spleen were purified by auto-MACS (Miltenyi Biotec, Bergisch Gladbach, Germany) and used as memory Th2 cells. For the Akt inhibitor treatment, memory Th2 cells were cultured with IL-7 (10 U ml^−1^) in the presence or absence of Akt inhibitor XI (10 μM) for 2 days.

### Identification of Gata3 phosphorylation sites

Flag-tagged cDNA for *Gata3* was inserted into a multi-cloning site of the CS-CDF-EG-internal ribosome entry site (IRES)-green fluorescent protein (GFP)-PRE vector. The lentivirus vector-containing culture supernatant was prepared as described previously^48^. D10G4.1 cells were infected with either mock control (CS-CDF-Eα-IRES-GFP-PRE) or Flag-Gata3 (CS-CDF-Eα-Flag-Gata3-IRES-GFP-PRE)-containing lentiviruses. Three days after infection, GFP-positive lentivirus-infected cells were sorted with a FACSAria machine (Becton Dickinson). Flag-tagged-Gata3 or mock-infected D10G4.1 cells were solubilized with the following protease inhibitor-containing IP buffer: 50 mM Tris-HCl (pH 7.5), 150 mM NaCl, 10% glycerol, 0.1% Tween, 1 mM EDTA, 10 mM NaF, 1 mM DTT and a protease inhibitor cocktail (Roche Applied Science, Indianapolis, IN), and lysed on ice for 30 min with gentle shaking and sonication with a Misonix sonicator (Misonix, Farmingdale, NY). The insoluble materials were removed by centrifugation and immunoprecipitation with anti-Flag M2 agarose (Sigma-Aldrich, St Louis, MO) was performed overnight at 4 °C. Immune complexes were eluted from the agarose by 3xFlag peptide, and the eluted Gata3 complexes were immunoprecipitated with an anti-Gata3 mAb (HG3-31, Santa Cruz Biotechnology, Inc. Santa Cruz, CA) or control IgG coupled to protein A/G beads. Immune complexes were eluted from the beads with elution buffer (50 mM Tris-HCl pH 8.0, 1% SDS, 10 mM EDTA) and separated by SDS–PAGE. The bands were excised from the gel and subjected to a mass spectrometric analysis to identify corresponding proteins. The gel pieces were washed twice with 100 mM bicarbonate in acetonitrile and the proteins were digested with trypsin. After adding 0.1% formic acid to the supernatant, the peptides were analysed by LC-MS/MS with a LTQ Mass Spectrometer (Thermo Scientific, Waltham, MA). The resulting MS/MS data set was analysed using the Mascot software programme (Matrix Science, Boston, MA).

### Immunoprecipitation and immunoblotting

Anti-Flag mAb (M2, Sigma-Aldrich), anti-Myc mAb (PL14) and anti-phospho Gata3 Ab (ab61052, Abcam, Cambridge, MA) were used for immunoprecipitation. Protein extracts from the transfected cells were prepared using RIPA buffer (1% NP-40, 0.25% Na-deoxycholate, 150 mM NaCl, 1 mM EDTA, 1 mM PMSF, 1 μg ml^−1^ of aprotinin, leupeptin and pepstain, 1 mM Na_3_VO_4_, 1 mM NaF and Tris-HCl, 50 mM, pH 7.4). For immunoprecipitation analyses, the cell lysates were subjected to a pre-clear process with protein G-sepharose at 4 °C for 1 h with rotation. The pre-cleared extracts were then subjected to immunoprecipitation with an anti-Flag mAb or an anti-phospho Gata3 Ab at 4 °C for 2 h, and then the immunocomplexes were precipitated with protein G-sepharose beads at 4 °C for 1 h. For immunoblot analyses, immunoprecipitates were eluted from beads in SDS-gel-loading buffer and run on 10% or 8–16% gradient polyacrylamide gels. After electrophoresis, the proteins were subjected to immunoblotting. The antibodies used for the Immunoblot analyses were anti-Gata3 (1 μg ml^−1^, HG3-31), anti-phospho Gata3 Ab (1 μg ml^−1^, ab61052, Abcam), anti-Flag (1 μg ml^−1^, M2), anti-Myc (1 μg ml^−1^, PL14), anti-Lamin A/C (1 μg ml^−1^, H-110, Santa Cruz Biotechnology, Inc.) and anti-HA (1 μg ml^−1^, 3F10, Roche, Basel, Switzerland). Images have been cropped for presentation. Full-size images are presented in Supplementary Fig. 1.

### Retroviral vectors and infection

The retrovirus vector, pMXs-IRES-GFP, was provided by Dr T. Kitamura (The University of Tokyo, Tokyo, Japan). The methods used to generate the virus-supernatant and for infection were described previously^49^. Infected cells were collected 5 days after stimulation and subjected to a quantitative reverse transcription–PCR (RT–qPCR) analysis and intracellular staining with anti-IL-4, anti-IFNγ and anti-Akt1.

### Quantitative RT–PCR

Total RNA was isolated using TRIzol reagent (Invitrogen, Waltham, MA). Reverse transcription was performed using the Superscript II kit (Invitrogen). For quantitative real-time PCR, a TaqMan universal PCR master mix was used for all reactions (Applied Biosystems, Waltham, MA) with an ABI Prism 7500 Sequence Detection System. Primers and probes used for the detection of *Tbx21*, *Il4*, *Ifng* and *Hprt* were described previously^5^. The data are shown as the relative expression normalized to *Hprt* signal.

### Chromatin immunoprecipitation assay

ChIP assays were performed using anti-Gata3 (D-16, Santa Cruz Biotechnology, Inc.), anti-p300 (N-15, Santa Cruz Biotechnology, Inc.) and Hdac2 (ab12169, Abcam) antibodies as described previously^33^. Quantitative-PCR analyses were performed on an ABI prism 7500 real-time PCR machine with probes from the Roche Universal Probe Library System. The specific primers and TaqMan probes used in this report were described previously^4^.

### Expression plasmids

Flag-tagged Gata3 (pFlag-CMV2-Gata3) mutants were generated by a PCR-based method. Myc-tagged N-terminal myristoylation and dn Akt1 were purchased from Millipore (Temecula, CA). Expression plasmids were transfected into 293T cells using FuGENE reagent (Roche) according to the manufacturer's protocol.

### *In vitro* kinase assay

Flag-tagged Gata3 complexes were immunopurified from 293T cells. Immunoprecipitates were incubated at 30 °C for 30 min in 40 mM HEPES (pH 7.5), 15 mM MgCl2, 0.1 mM EDTA, 5 mM 2-mercaptoethanol (2-ME), 25 μM cold ATP and 5 μCi of [γ-^32^P]ATP. Phosphorylated Gata3 was analysed by SDS–PAGE and autoradiography.

### In-gel kinase assay

Flag-tagged Gata3 complexes from 293T cells were run on a 12% SDS–PAGE gel with the resolving gel containing 0.02% recombinant GST-Gata3 or myelin basic protein as a negative control. After the electrophoretic run, the gel was washed with 50 mM Tris-HCl (pH 8.0), 20% 2-propanol and 50 mM Tris-HCl (pH 8.0) and 5 mM 2-ME. The proteins were subsequently denatured in 50 mM Tris-HCl (pH 8.0), 5 mM 2-ME, and 8 M guanidinium hydrochloride for 2 h. Proteins were then slowly renatured over a period of 16 h with six changes in a buffer containing 50 mM Tris-HCl (pH 8.0), 5 mM 2-ME and 0.04% Tween 20. The gel was equilibrated in a buffer containing 40 mM HEPES (pH 7.5), 15 mM MgCl_2_, 0.1 mM EDTA, 5 mM 2-ME for 90 min and then incubated in the same buffer containing 10 μCi of [γ-^32^P]ATP. After washing, the gel was dried and subjected to autoradiography.

### Preparation of human memory CD4 T cells

Whole blood was obtained from four healthy donor volunteers. The protocol was approved by the Institutional Ethics Committee (no. 1016). peripheral blood mononuclear cells were isolated by Ficoll-Paque (Pharmacia-Upjohn, Uppsala, Sweden) gradient centrifugation. CD45RO^+^ memory CD4 T cells were purified using the memory CD4^+^ T-cell isolation kit (Miltenyi Biotec) and auto-MACS (Miltenyi Biotec). Then, cells were stimulated with PMA (10 ng ml^−1^) and ionomycin (500 nM) for 4 h and subjected to intracellular staining with anti-IL-4, anti-GATA3, anti-phospho GATA3, anti-T-BET and anti-IFNγ.

## Additional information

**How to cite this article:** Hosokawa, H. *et al*. Akt1-mediated Gata3 phosphorylation controls the repression of IFNγ in memory-type Th2 cells. *Nat. Commun.* 7:11289 doi: 10.1038/ncomms11289 (2016).

## Supplementary Material

Supplementary Information Supplementary Figure 1
