# Supplementary material for: Akt1-mediated Gata3 phosphorylation controls the repression of IFNγ in memory-type Th2 cells
Source: Nat Commun. 2016 Apr 7;7:11289. doi: 10.1038/ncomms11289 (PMC4829694; doi:10.1038/ncomms11289)

Supplementary Figure 1
uncropped blots

Fig. 1a

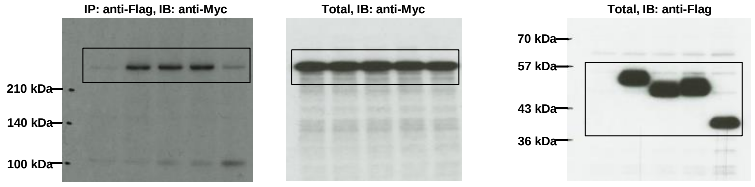

Fig. 1c

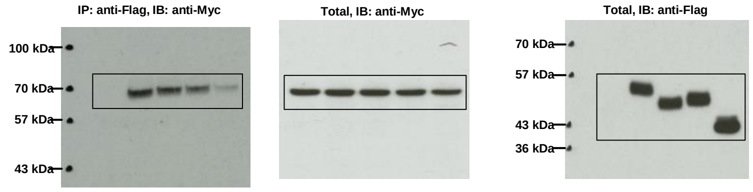

Fig. 2a

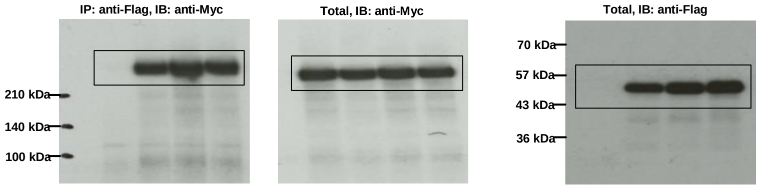

Fig. 2b

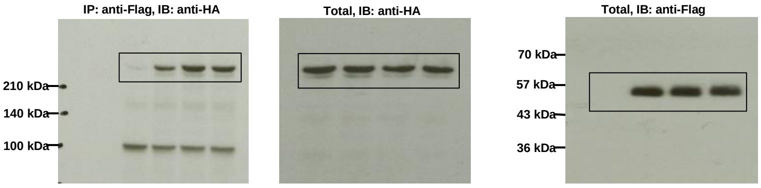

Fig. 2c

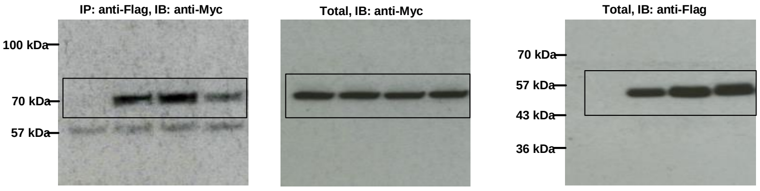

Fig. 2d

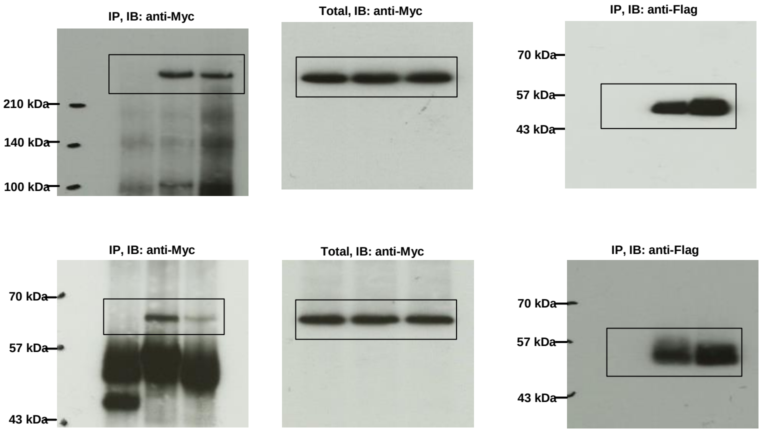

Fig. 2e

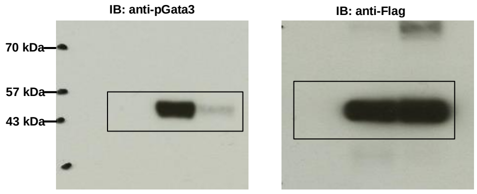

Supplementary Figure 1 (continued)

Fig. 4d

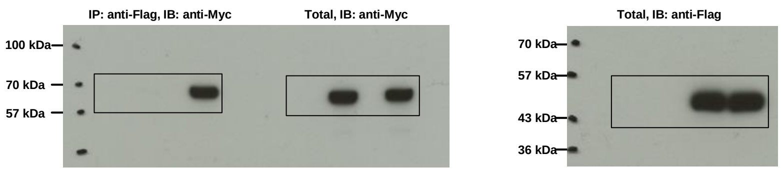

Fig. 4e

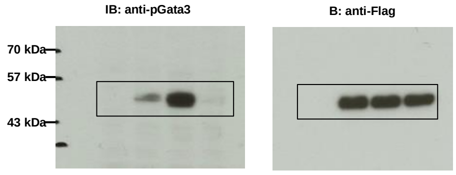

Fig. 4g

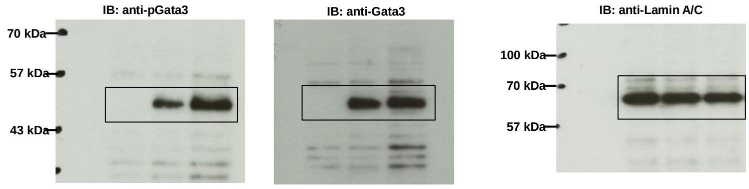

Fig. 4h

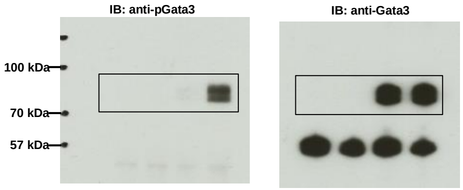

Fig. 4i

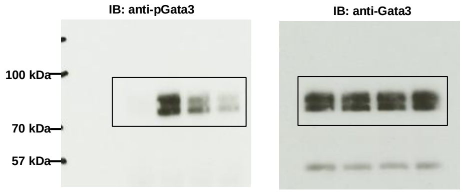

Fig. 5c

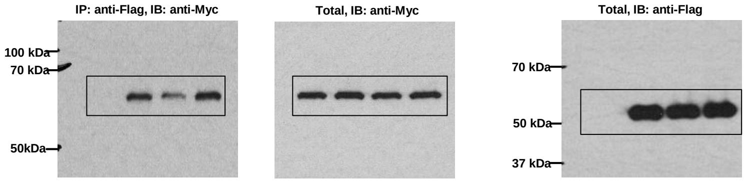

Supplement: Supplementary Information — Supplementary Figure 1 [file ncomms11289-s1.pdf]
